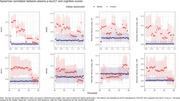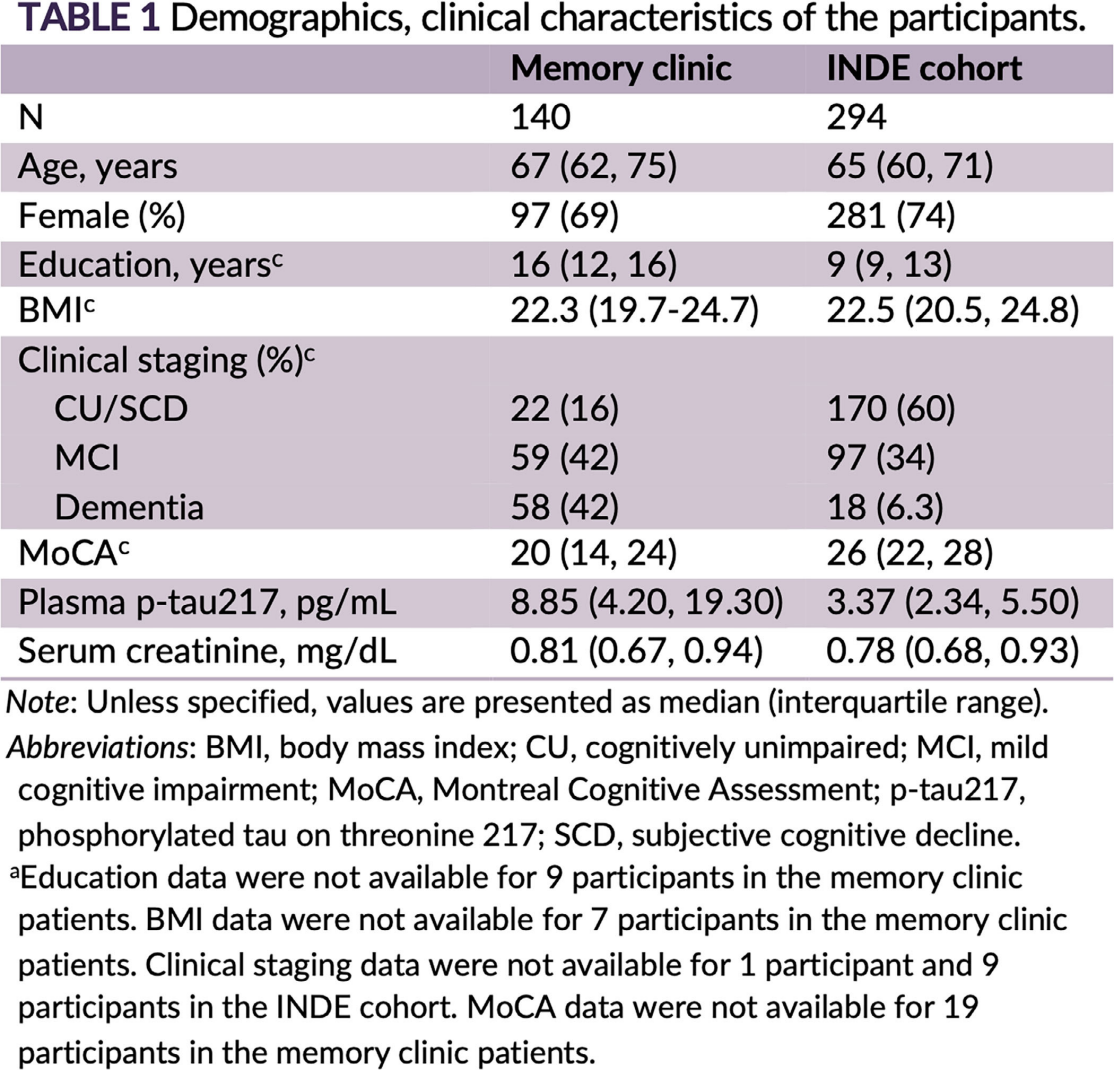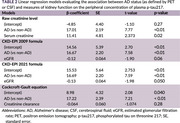# Exploring kidney function thereshole for plasma *p*‐tau 217 use

**DOI:** 10.1002/alz70856_105379

**Published:** 2026-01-09

**Authors:** Thanaporn Haethaisong, Adipa Chongsuksantikul, Prawit Oangkhana, Watayuth Luechaipanit, Thanakit Pongpitakmetha, Poosanu Thanapornsangsuth

**Affiliations:** ^1^ Thai Red Cross Emerging Infectious Diseases Health Science Centre, King Chulalongkorn Memorial Hospital, The Thai Red Cross Society, Bangkok, Thailand; ^2^ Thai Red Cross Emerging Infectious Diseases Health Science Centre, King Chulalongkorn Memorial Hospital, Bangkok, Thailand; ^3^ Department of Pharmacology, Faculty of Medicine, Chulalongkorn University, Bangkok, Thailand; ^4^ Division of Neurology, Department of Medicine, Faculty of Medicine, Chulalongkorn University, Bangkok, Thailand; ^5^ Chula Neuroscience Center, King Chulalongkorn Memorial Hospital, The Thai Red Cross Society, Bangkok, Thailand; ^6^ Memory Clinic, King Chulalongkorn Memorial Hospital, The Thai Red Cross Society, Bangkok, Thailand

## Abstract

**Background:**

Plasma tau phosphorylated at Thr217 (*p*‐tau217) is emerging as a core biomarker for Alzheimer's disease (AD), with high diagnostic accuracy and prognostic value. However, impaired kidney function may lead to elevated plasma *p*‐tau217 unrelated to AD pathology, potentially limiting its utility in individuals with kidney disease. It remains unclear which kidney function parameter and threshold should be used to determine whether plasma *p*‐tau217 reliably reflects AD pathology.

**Method:**

We analyzed data from patients evaluated at the Memory Clinic, King Chulalongkorn Memorial Hospital, and the Neurology Clinic, Neurological Institute of Thailand (2022–2024), who had confirmed AD status via florbetaben PET or CSF Aβ42/p‐tau. Plasma *p*‐tau217 was modeled as a dependent variable in multivariable linear regression, incorporating AD status and kidney function. Kidney function was assessed using serum creatinine, estimated glomerular filtration rate (eGFR) from two CKD‐EPI equations, and creatinine clearance (Cockcroft‐Gault). The parameter with the largest effect size was selected for threshold analysis. To identify a kidney function threshold beyond which plasma *p*‐tau217 becomes less reliable, we analyzed participants from the INDE cognitive aging cohort (NCT06375213). Participants were stratified by kidney function thresholds, and for each threshold, 10 participants were resampled with replacement 1,000 times. We calculated Spearman correlations between plasma *p*‐tau217 and cognitive scores (MoCA, MMSE, and Wechsler Memory Scale LM) to identify the threshold at which this relationship weakens.

**Result:**

Among 140 patients with PET or CSF‐confirmed AD, plasma concentration of *p*‐tau217 was significantly influenced by kidney function in models incorporating serum creatinine and eGFR (CKD‐EPI 2021) (Table 2). Threshold analysis sampled from 294 INDE participants. When eGFR fell below 57 mL/min/1.73 m^2^, the correlation between plasma *p*‐tau217 and MoCA, MMSE, LMII weakened (Figure 1). No clear trend was observed when classifying kidney function using serum creatinine level.

**Conclusion:**

eGFR (CKD‐EPI 2021) appears to be the most appropriate measure for assessing kidney dysfunction when interpreting plasma *p*‐tau217. A threshold of 57 mL/min/1.73 m^2^ may indicate when kidney impairment begins to confound its relationship with cognitive function. Further studies should examine individuals with more severe kidney impairment and alternative kidney biomarkers, such as cystatin C.